# Forward-time simulation of realistic samples for genome-wide association studies

**DOI:** 10.1186/1471-2105-11-442

**Published:** 2010-09-01

**Authors:** Bo Peng, Christopher I Amos

**Affiliations:** 1 Department of Epidemiology, The University of Texas M. D. Anderson Cancer Center, Houston, Texas, 77030, USA

## Abstract

**Background:**

Forward-time simulations have unique advantages in power and flexibility for the simulation of genetic samples of complex human diseases because they can closely mimic the evolution of human populations carrying these diseases. However, a number of methodological and computational constraints have prevented the power of this simulation method from being fully explored in existing forward-time simulation methods.

**Results:**

Using a general-purpose forward-time population genetics simulation environment, we developed a forward-time simulation method that can be used to simulate realistic samples for genome-wide association studies. We examined the properties of this simulation method by comparing simulated samples with real data and demonstrated its wide applicability using four examples, including a simulation of case-control samples with a disease caused by multiple interacting genetic and environmental factors, a simulation of trio families affected by a disease-predisposing allele that had been subjected to either slow or rapid selective sweep, and a simulation of a structured population resulting from recent population admixture.

**Conclusions:**

Our algorithm simulates populations that closely resemble the complex structure of the human genome, while allows the introduction of signals of natural selection. Because of its flexibility to generate different types of samples with arbitrary disease or quantitative trait models, this simulation method can simulate realistic samples to evaluate the performance of a wide variety of statistical gene mapping methods for genome-wide association studies.

## Background

Simulated data sets of known disease-predisposing loci (DPL) have been widely used in the development and application of statistical methods that detect susceptibility genes for human genetic diseases [1,2]. Whereas simple samples simulated under idealized assumptions can be used to validate properties of statistical gene mapping methods, only samples that reflect the complex structure of the human genome and the genetic basis of human genetic diseases can be used to evaluate and compare the statistical power of these methods and to compare various sampling designs under realistic conditions. Otherwise, a gene mapping method may perform well in theory and on simulated datasets, but poorly on real datasets [3,4].

Thanks to rapid advances in genotyping technology, genome-wide association studies (GWAS) have been increasingly used to decipher multiple interacting genetic and environmental factors that are responsible for complex human diseases [5,6]. The increased use of GWAS has fostered the development of statistical methods and software applications for the simulation of genetic samples with high-density markers over long genome regions [7]. Such simulations have been used to compare the power of popular study designs and statistical methods for GWA studies [8-10], to simulate case-control samples for evaluating the power of new statistical methods [11], and to study the performance of statistical tests under different disease models [12,13].

Several programs have been developed to simulate genetic data for GWAS. Excluding specialized methods that simulate genetic data for particular types of samples, currently available simulation methods can be categorized roughly as resampling-based [9,14,15], backward-time-based (coalescent) [16-18], and forward-time-based [19-21]. Resampling methods permute or sample from existing genome sequences. Although these methods excel at retaining allele frequency and linkage disequilibrium (LD) information from existing sequences, they are limited in their ability to introduce new genetic features (such as the effects of natural selection) and new haplotypes. For example, from a sample of 20,000 simulated sequences of 40 tightly linked markers over a 100-kbp region on chromosome 17 that we simulated using HAPGEN [9], only 74 unique haplotypes exist because all the haplotypes are derived from the 63 unique haplotypes that exist in the European HapMap sample (CEU) [22] using an imputation approach.

Coalescent methods excel at simulating random samples, but it is difficult to use these methods to simulate case-control or other types of samples with genetic diseases because a coalescent simulation constructs a genealogical tree from samples with unknown genotypes and cannot effectively control the number of affected individuals once the genotypes of these samples are simulated [23,24]. If a large number of samples are simulated before a disease model is applied, the coalescent method becomes inefficient, especially when long genomic sequences are simulated, unless special algorithms are used to approximate the standard coalescent process [25-27]. In addition, many coalescent method-based programs simulate samples with random marker locations, which makes defining a genetic disease with consistent DPL difficult. Finally, most of these methods were designed to simulate case-control samples of relatively simple disease models and therefore have limited applicability to important research areas such as the detection of gene-environment interaction, admixture mapping, or family-based associations.

Forward-time simulation methods evolve a population forward in time, subject to arbitrary genetic and demographic factors. Because such a simulation can closely mimic the complex evolutionary histories of human populations that harbor the genetic diseases of interest, these methods can, in theory, simulate genetic samples with arbitrary complexity. Arbitrary disease models could be applied to the resulting population from which samples based on different study designs can be drawn and analyzed [21]. However, this method is inefficient because ancestors who do not have offspring in the resulting population are simulated, and a large population must be simulated before samples can be drawn from it. In addition, the properties of populations simulated using a forward-time approach depend heavily on the initial populations, which are often simulated under arbitrary equilibrium assumptions. Even if the same initial populations are used, the resulting populations will vary because of random genetic drift. Finally, existing implementations of forward-time simulations [19-21,28] vary in their abilities to simulate high-density genetic markers with realistic LD patterns and none of them can readily simulate samples that use existing genetic markers in the human genome.

This paper presents a forward-time simulation approach that addresses most of these problems. To retain the complex genetic structure of human populations, this algorithm creates an initial population of selected markers from a real sample. It then evolves this population forward-in-time, subject to mutation, recombination, natural selection, and rapid population expansion. This process uses an optional scaling algorithm to improve its performance when weak additive selection forces are used and uses a trajectory-simulation method to control the frequency of disease-predisposing alleles (DPAs). Depending on specific applications, the last step of this process involves different post-processing steps. For example, a rejection-sampling algorithm can be used to simulate case control samples or trio families (affected offspring with parents) with rare diseases. We validated the properties of this simulation method by comparing simulated samples with real data and demonstrated the wide applicability of this simulation method using four examples, including a simulation of case-control samples with a disease caused by multiple interacting genetic and environmental factors, a simulation of trio families affected by a DPA that has been subject to either slow or rapid selective sweep, and a simulation of a structured population resulting from a recent population admixture. The new simulation approach and all examples were implemented using a general-purpose forward-time population genetics simulation environment simuPOP [29], which has been used to implement other forward-time simulations [21,30].

## Methods

Because of the complexity of human genomes and their largely unknown evolutionary histories, it is infeasible to simulate samples that closely resemble human populations by evolving a simulated initial population. Therefore, our simulation method uses real empirical data sets to simulate large populations with additional genetic variations while retaining key features of the empirical data sets. Thanks to rapid advances in genotyping technology, the genotype data of millions of single nucleotide polymorphism (SNP) markers of hundreds or even thousands of individuals are currently available [6,31], and higher density data will become available in the near future [32]. The availability of data facilitates the creation of an initial population with selected markers that match an existing sample, which usually contains markers from commercially available genotyping platforms.

The first step of our simulation method is to create an initial population from a real sample with selected markers. Depending on the application, one may want to start from an existing GWA study with thousands of controls, such as the control data from the Wellcome Trust Case Control Consortium [6], or from a publicly available data set, such as Phase 2 or Phase 3 of the HapMap data set [31]. Our study used 993 unrelated individuals (parents in trio and duo samples and all unrelated individuals) in 10 populations of the Phase 3 HapMap data set because these data are readily available. Depending on the specific application, markers can be chosen according to markers used in real-world studies (e.g., the markers on the Illumina 550k genotyping chip) or by marker distance and minor allele frequency; individuals from one or more HapMap populations can be selected either as separate populations or as a single population.

We consider the initial populations as small, isolated populations before the expansion of a typical human population (around 12,000 years or 600 generations ago, if we assume 20 years per generation with the invention of agriculture) [33]. We then expand these populations linearly to a larger population of 10^5 ^individuals by adding the same number of individuals each year, subject to mutation, recombination, and natural selection. We use linear population expansion instead of a more commonly used exponential expansion model because a linear model expands the initial population faster at first, thus better preserving genetic diversity in the initial population and resulting in a final population with a larger effective population size. For example, if we start with 993 individuals from the Phase 3 HapMap sample and expand the population for 500 generations using linear and exponential population expansion models, the effective population sizes of the expanded populations with 10^5 ^individuals would be 12,658 and 4603, respectively [34]. The former is comparable to the effective population size of real human populations.

During evolution, we mutate all SNP markers according to a symmetric diallelic mutation model with a mutation rate of 10^-8 ^per basepair per generation. At each generation, parents are chosen at random and pass their genotypes to offspring according to Mendelian laws. Parental chromosomes are recombined according to a fine-scale genetic map estimated from the HapMap data set [35] before one of the recombinants is passed to an offspring. If a selection model is specified, parents are chosen with probabilities that are proportional to their relative fitness values. Our simulation method supports both single-locus and multilocus natural selection models, including models that involve multiple interacting DPL. If multiple populations are simulated, a stepping stone migration model with a low migration rate is applied to control the genetic distance between the populations [36].

A scaling approach is used to improve the efficiency of our simulation [37]. Compared to a regular simulation that evolves a population of size *N *for *t *generations, a scaled simulation with a scaling factor λ evolves a smaller population of size *N/*λ for *t*/λ generations with magnified (multiplied by λ) mutation, recombination, and selection forces. This method could be justified by a diffusion approximation to the standard Wright-Fisher process [34,37]; however, because the diffusion approximation only applies to weak genetic forces in the evolution of haploid sequences, it cannot be used when nonadditive diploid or strong genetic forces are used. Our simulation program simulates populations with specified population size so a population simulated using a scaling factor λ would be comparable to an unscaled simulation of a population that is λ times larger.

To simulate a genetic disease, we control the frequencies of DPAs at DPL using presimulated allele frequency trajectories [21,38]. Either a forward-time approach or a backward-time approach can be applied. More specifically, if we assume that a DPA existed before population expansion, we simulate the frequency of the DPA forward in time until it reaches the present generation. The simulation starts from the frequency of the DPA in the initial population and is restarted if the allele frequency at the present generation falls out of the desired range [24]. If the mutant is recent (e.g., appears within the past 500 generations), we simulate from the frequency of the DPA at the present generation backward in time until the allele gets lost. Multilocus natural selection models are supported with the restriction that DPAs have to be unlinked. After the allele frequency trajectories of DPAs are simulated, we use a special random mating scheme to evolve the population forward in time while following the simulated trajectories at these loci [21]. This mating scheme simulates large intermediate populations and forms offspring populations with desired allele frequencies by selecting offspring according to their genotypes at controlled loci.

The final postprocessing step of the simulation process will vary depending on the individual application. To simulate a common disease with enough affected individuals in the simulated population, we can draw samples directly from the population after the affection status of each individual is determined, usually using a penetrance model that yields the probability that an individual is affected with a disease according to his or her genotype (Pr(affection status | genotype)). Alternatively, a rejection-sampling algorithm could be used to draw case-control samples or samples with independent offspring (such as trios) of a rare disease. More specifically, we choose parents from the simulated population and produce offspring repeatedly, apply the penetrance model to determine the affection status of each offspring, and continue the process until enough samples are collected.

We implemented the proposed simulation method using several simuPOP scripts [29], including scripts to download and select markers from Phase 2 and Phase 3 of the HapMap data set, a main script (simuGWAS.py) to evolve a population forward in time with customizable demographic and genetic features, and postprocessing scripts for the examples presented in this article. The use of scripts in this implementation makes it easy to adapt the examples for other simulations or even to customize the evolutionary process using alternative genetic features. The amount of time required to perform a simulation depends on the size of the simulation and scales roughly linearly with the number of markers and individuals. For example, the simulations in example 1 (5000 markers, using a scaling factor of 2 with a final population size of 25000) and example 3 (500 markers, unscaled) took 31 and 14 minutes, respectively, on a Macintosh workstation with a 2.26-G Intel Xeon processor and 8 G of random access memory.

## Results

### Example 1: Typical simulations with or without scaling

We created an initial population with the 993 independent individuals of the HapMap Phase 3 data set, using 5000 markers on region 2p16.3 (chr2:51002576-60032817). This region spans 9.03 Mbp with a genetic distance of 6.97 cM. It contains the ENr112 ENCODE region and has an average marker distance of 1.81 kb. We evolved this population for 500 generations until it reached 50,000 individuals, subject to mutation (at a mutation rate of 10^-8 ^per locus per generation), recombination (according to the genetic distance between adjacent markers), no selection, and linear population expansion.

**Figure 1:**
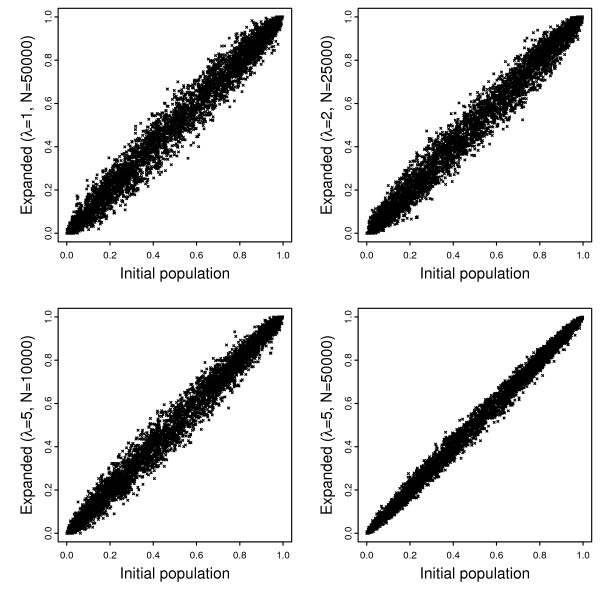
**Allele frequencies of the initial (*x*-axis) and expanded (*y*-axis) populations of four simulations with populations sizes 50000, 25000, 10000 and 50000, and scaling factors λ = 1 (unscaled), 2, 5 and 5 respectively**.

In order to evaluate the quality of simulated populations and the impact of the scaling technique, we simulated three expanded populations of sizes 50000, 25000, and 10000 using scaling factors 1 (unscaled), 2, and 5 respectively, and an expanded population of 50000 individuals using a scaling factor of 5. Whereas the first three populations are scaled versions of the same evolutionary process, the last one is comparable to an unscaled simulation of a population of size 250000. Compared to the first three simulations, genetic drift has a smaller impact on the last simulation because of its larger population sizes during evolution. This is demonstrated in Figure 1 where the allele frequencies at 5000 markers for all simulated populations are compared with those of the initial population.

**Figure 2:**
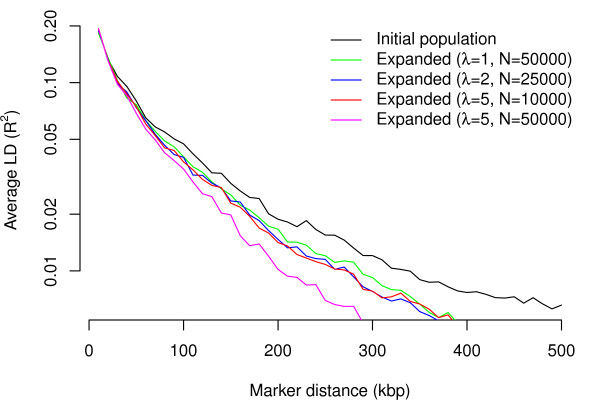
**Average LD values as a function of marker distance for the initial population and four expanded populations of sizes 50000, 25000, 10000 and 50000, using scaling factors λ = 1 (unscaled), 2, 5, and 5 respectively**. The y-axis is plotted in log scale to distinguish LD curves in low LD regions. Marker distances were cut into bins of 10 kbp. For example, the average LD at point 200 kbp represents the mean pairwise LD values of all pairs of markers that were 200 kbp to 210 kbp apart.

The evolution of LD in such an evolutionary process is more complicated. According to Figure 2, all simulated populations had lower LD than those of the initial population. Although populations simulated using a scaling approach tended to have lower LD than those from unscaled simulations, the differences between mean R^2 ^values were negligible especially for markers that are less than 200kbp apart. A more detailed analysis showed that average LD increased and then decreased during the evolutionary process of all simulations. This phenomenon could be explained by the fact that our simulation started from a relatively small population, so LD first built up because of a bottleneck effect. With increasing population size, the natural decay of LD through genetic recombination gradually prevailed at a rate accelerated by the impact of rapid population expansion [39,40]. The simulation with a scaling factor of 5 and population size 50000 had the lowest LD values because it had a relatively short period of bottleneck and a faster rate population expansion than other simulations.

### Example 2: A genetic disease with two DPL

We extracted 6000 markers (2000 markers each) on chromosomes 2 (chr2:20014298-31200250), 5 (chr5:20005983-32781509) and 10 (chr10:41756307-55305682) of 993 independent individuals from Phase 3 of the HapMap sample. We selected markers from a commercially available genotyping chip (the Illumina 550k array) to match markers used in real-world GWAS. The average distance between adjacent markers was 5.61 kb. Two markers, rs4491689 (chr2:26494285) and rs6869003 (chr5:27397573), were selected to be the DPL of a genetic disease. The first marker was assumed to be under purifying selection, with fitness values of 1, 0.996, and 0.994 for genotypes *AA*, *AG*, and *GG*, respectively. The second marker was assumed to be under positive selection, with fitness values of 1, 1.001, and 1.005 for genotypes *CC*, *CT*, and *TT*, respectively. A multiplicative multilocus selection model was used. Because these two loci reside on different chromosomes, we considered natural selection to be applied to these loci independently [41]. We assumed that the DPAs existed longer than 500 generations, and we used a forward-time simulation method to simulate trajectories of the frequencies of the minor alleles at both loci, starting from their frequencies in the chosen HapMap sample (0.28 for marker rs4491689 and 0.07 for marker rs6869003). The ending allele frequencies were 0.05 for marker rs4491689 and 0.15 for marker rs6869003, which were chosen in concordance with the selection pressure that was applied to each marker. We did not scale this simulation because of the use of a nonadditive diploid selection model.

We used a logistic model with gene-gene and gene-environment interactions to model a disease that involves these two genetic markers and a random environmental factor with two states 0 and 1. We assumed that the disease was mild and was not the source of the selection pressure on the two DPL. The model can be expressed as logit (Pr(Y = 1|g_1_,g_2_,e)) = *α+β*_1_*g*_1_+*β*_2_*g*_2_+*β*_3_g_1_g_2_+γ_1_g_1_*e*+γ_1_g_1_*e *+ γ_2_*g*_2_*e *where *Y *is the disease status, *g*_*1 *_and *g*_2 _are number of DPAs at two markers respectively, and *e *is the random environmental factor. This model is an extension of the one-gene, one-environmental model used in Li and Conti [42]. We chose positive*α*, *β*_*i*_, and γ_*i *_values so that the presence of each DPA increases the probability that an individual is affected with the disease. We chose *β*_1 _= *β*_2_/2, γ_1 _= γ_2_/2, so that the DPA at marker rs4491689 had less impact on the disease than the DPA at marker rs6869003. Finally, we controlled parameters α, *β*_*i*_, and *γ*_*i *_so that the prevalence of the disease was 1%. Because there were less than 1000 affected individuals in the expanded population, we used a rejection-sampling algorithm to populate an offspring population with exactly 1000 cases and 1000 controls from the expanded population.

**Figure 3:**
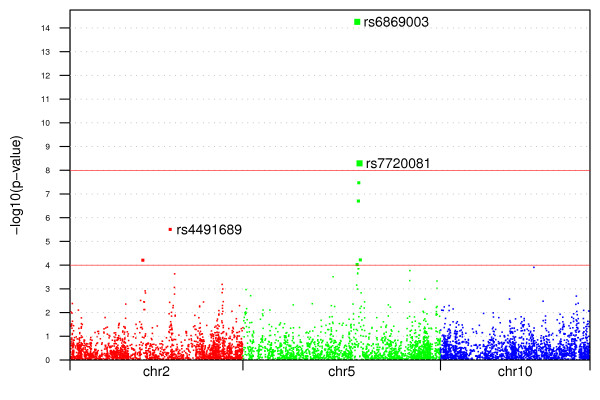
**Negative of the base 10 logarithm of *p*-values of allele-based χ^2 ^tests between 1000 cases and 1000 controls at 6000 markers (2000 each) on chromosomes 2, 5, and 10**. Markers rs4491689 and rs6869003 are causal. Marker rs7720081 has low *p*-value because it is closely linked to marker rs6869003.

We counted the number of alleles in cases and controls, created a 2 by 2 contingency table and used a χ^2 ^test to test the association between the disease status and alleles at each marker. The negative of the base 10 logarithm of the *p*-values at all markers were plotted in Figure 3. Although the χ^2 ^tests detected both genetic factors correctly, more sophisticated statistical methods or larger samples would be needed to detect the gene-gene and gene-environment interactions in this data set.

### Example 3: Simulations of slow and rapid selective sweep

In Example 2, natural selection was not expected to have a strong impact on LD patterns around DPL because it changed frequencies of both DPAs slowly over a long period of time. In contrast, strong selection can bring a new beneficial mutation to high frequency or fixation in a population in a relatively short period of time. This phenomenon, also called selective sweep, has a profound impact on patterns of linked genetic variation through the hitchhiking effect. The signatures of selective sweep have been used for the development of statistical methods to identify chromosomal regions that have been under positive selection [43] and for the identification of DPL in genome wide association analysis [44]. Although theoretical models of selective sweep have been simulated for methodological development using coalescent approaches [43,45,46], explicit simulation of selective sweep using a forward-time approach can be used to study the impact of different levels of natural selection on different regions of the human genome and to produce realistic samples to assess the power of statistical methods.

In order to closely examine the impact of selective sweep on existing LD patterns of a chromosomal region, we extracted 500 markers on a short region on chromosome 2 (chr2:234157787-234573235) from 170 independent individuals from the JPT+CHB population of Phase 3 of the HapMap data set. We selected this region because it belongs to the ENr131 ENCODE region with a mean distance of 0.83 kb between markers. We selected marker rs2173746 with alleles *C *and *T *from one of the two haplotype blocks in this region and applied different levels of positive selection during the evolution of this population.

In our first simulation, we assumed that allele *T *at this marker was introduced more than 500 generations ago. This allele had a frequency of 5.88% in the initial HapMap population and reached a frequency of 99% after 500 generations due to positive natural selection with fitness 1, 1.02, and 1.03 for genotypes *CC*, *CT*, and *TT*, respectively. A forward-time trajectory simulation algorithm was used to control the frequency of allele *T *at the present generation. In our second simulation, we cleared allele *T *at this marker from the initial population and introduced it as a new mutant during the evolutionary process. Using a backward-time trajectory simulation process, an allele *T *was introduced at generation 268 and was brought to a frequency of 99% using a stronger force of natural selection with fitness 1, 1.05, and 1.11 for genotypes *CC*, *CT*, and *TT*, respectively.

We drew 1000 trios from the simulated populations using a rejection-sampling algorithm. More specifically, we repeatedly chose parents and produced offspring. We determined the affection status of each offspring using a logistic regression model logot(Pr(*Y_i _*= 1))= -0.5-*g_i_*, where *g_i _*is the number of allele *T *at locus rs2173746. We kept only affected offspring and their parents in the sample until 1000 trios were collected. We used LAMP [47] to analyze the data set.

**Figure 4:**
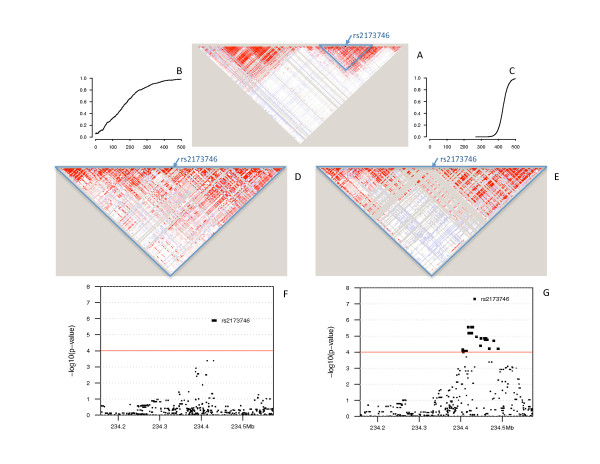
**An initial population of 170 independent individuals of the JPT+CHB population of Phase 3 of the HapMap data set was expanded to large populations and subjected to slow (B, D, F) and rapid (C, E, G) selective sweeps at locus rs2173746**. The trajectories of the frequencies of allele *T *at this marker in simulations after slow (B) and rapid (C) sweeps are plotted. The LD maps of 500 markers on chromosome 2 of the initial population (A), and 100 markers around locus rs2173746 of expanded populations after the slow (D) and rapid (E) sweeps are plotted. 1000 cases and 1000 controls were drawn from these expanded populations. The negative of the base 10 logarithm of *p*-values at 500 markers are plotted for slow (F) and rapid (G) sweeps.

Figure 4 plots the simulated trajectories (Figure 4B and 4C) as well as LD maps, drawn by HaploView [48], of this region before evolution for all 500 markers (Figure 4A) and after evolution with slow (Figure 4D) and rapid (Figure 4E) sweeps for 100 markers around the DPL. The LD maps plot the pairwise D' measure of LD between all markers in a region, with high LD pairs marked in bright red. Comparing the LD maps around the DPL before (Figure 4A) and after evolution (Figure 4D and 4E), it was clear that rapid selective sweep introduced blocks of monomorphic markers (gray areas around marker rs2173746 in Figure 4E) when the haplotype with the mutant became prevalent when the mutant (allele *T *at marker rs2173746) was brought to a high frequency (99%). In contrast, the impact of slow selective sweep on the LD patterns around the DPL was barely discernible. As a matter of fact, the initial population had 50 haplotypes over a region of 100 markers around marker rs2173746 (50 markers on each side), with a frequency of 16% for the most popular haplotype. After rapid selective sweep, only 19 haplotypes existed in this region, with a frequency of 97% for the most popular haplotype. In contrast, 195 haplotypes were present in the population resulting from slow selective sweep, with a frequency of 44% for the most popular haplotype.

The simulated populations could be used to test the performance of statistical tests designed to detect signals of positive selection along the human genome [43] and to detect DPL if the DPA was under positive selection [44]. For example, when we applied family-based association tests to two samples of trio families drawn from the simulated populations, the signals from rapid selective sweep (Figure 4G) appeared to be wider than those from slow selective sweep (Figure 4F). This phenomenon could be explained by stronger LD between the DPL and its surrounding loci for the simulation with rapid selective sweep, but a quantitative analysis using a large number of simulations would be needed to draw a definitive conclusion.

### Example 4: Simulation of admixed populations

Population structure has been known to cause spurious associations in case-control association studies [49], so several statistical methods have been developed to reduce the impact of population structure on GWA studies [50-52]. On the other hand, population admixture causes long-range admixture LD that could be used to map diseases in admixed populations [53,54]. Although simulations have been used to evaluate the performance of these statistical methods, they have not been complex enough to challenge the statistical methods under realistic situations [3]. For example, Pfaff et al. [55] broke existing LD of the founder populations from HapMap samples by sampling alleles instead of haplotypes so that only admixed LD existed in the simulated sample.

We aimed to simulate realistically admixed populations by mixing populations simulated from the HapMap populations. We extracted 5000 markers with minor allele frequency greater than or equal to 0.05 from chromosome 2 (chr2:50002476-60382263) using 170 and 143 independent individuals from the Phase 2 of the HapMap Japanese in Tokyo, Japan and Han Chinese in Beijing, China (JPT+CHB) and Maasai in Kinyawa, Kenya (MKK) populations. The two populations were expanded to a total size of 50,000 individuals. A low-level migration rate of 0.0001 was applied to keep the genetic distance between these two populations around its original level of 0.11 (measured using *F_ST_*) [56].

To control the levels of true LD and admixture LD, we mixed large populations to avoid elevated LD caused by a founder effect. We mixed these two populations using a continuous gene flow model where 5% of individuals from the MKK population migrated to the JPT+CHB population for 10 generations [57]. At the beginning of population mixing, we assigned an ancestral value of 0 to individuals from the JPT+CHB population and a value of 1 for individuals from the MKK population. During the admixture process, the offspring ancestral values were recorded as the mean of parental ancestral values. We used a positive assortative mating scheme to mix individuals because migrants usually do not mate randomly with natives during a real-world admixture process, and individuals would be efficiently mixed and have similar ancestral values after only a few generations if the standard Wright-Fisher random mating process were used to mix parents regardless of their ethnicity. More specifically, we divided individuals into two groups according to their ancestral value, one with ancestral values greater than or equal to 0.5 and another with ancestral values less than 0.5. During the population of an offspring generation, 80% of all mating events happened within the these two groups and the rest of the mating events happened with parents chosen randomly from the whole population [58]. This process slowed down the admixture process and resulted in a distribution of individual ancestry values that is closer to that of real populations, such as the mixture distribution of European ancestry among all African Americans [59].

**Figure 5:**
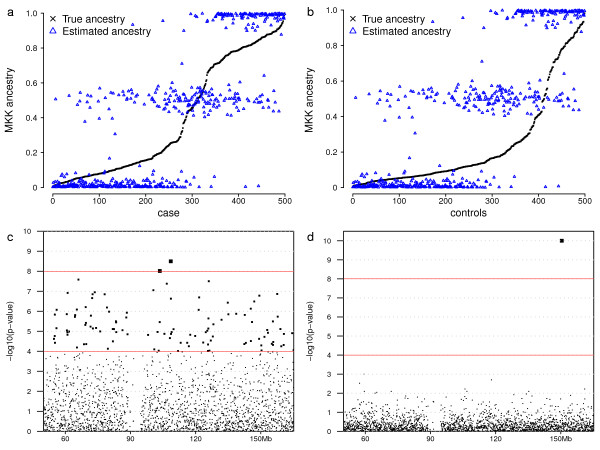
**Ancestry values and *p*-values of association tests**. The top figures plot recorded and estimated MKK ancestry values of 500 cases (a) and 500 controls (b). Individuals are sorted by their true MKK ancestry values. The bottom figures plot the negative of the base 10 logarithm of *p*-values of allele-based χ^2 ^tests (c) and structured association tests (d) between 500 cases and 500 controls at 2000 markers.

We applied to the population a penetrance model in which individuals' probability of being affected equals 0.05 + ancestry/6, where ancestry is the individual's MKK ancestry value. Individuals with higher MKK ancestry values were more susceptible to this disease, but none of the 5000 markers caused the disease directly. Because there were enough affected individuals in the simulated population, we drew 500 cases and 500 controls directly from the simulated population. We used the STRUCTURE program to estimate the ancestry values of cases and controls from their genotypes [50] and plotted the estimated MKK ancestries against the recorded ancestry values for each individual (Figure 5.a and 5.b). Because individuals with high MKK ancestry values are more likely to be affected, cases on average had higher MKK ancestry values than controls.

In order to demonstrate the impact of population structure on association analysis, we applied allele-based χ^2 ^tests and structured association tests proposed by Pritchard et al. [60] to detect the association between disease status and 2000 simulated markers (Figure 5.c and 5.d). Although the disease is not directly caused by any of the simulated markers, a large number of spurious associations were detected by the χ^2 ^tests. In contrast, the structured association tests estimated individual ancestry values to control the impact of population structure and successfully removed most spurious associations.

## Discussion

The genetic composition of a human population is the result of a long and complex evolutionary process. The demographic and genetic features of this process have profound implications in the mapping of susceptibility genes responsible for human genetic diseases [61]. Whereas resampling-based methods capture the complexity of existing genome sequences with no control over the impact of additional genetic and demographic forces, and the coalescent methods simulate simple samples based solely on a few theoretical models, the forward-time simulation method described in this article retains key properties of human genomes by evolving a population from real human sequences while allowing the introduction of additional genetic forces such as natural selection. Because this method is not constrained by any theoretical limit, it can be used to simulate realistic samples for a variety of research topics for GWA studies.

In order to retain key features of real human genomes during evolution, this method expands the founder population rapidly. Because the size of the founder population is likely to be small, this evolutionary process currently suffers from a bottleneck effect during the initial stage of population expansion, resulting in a loss of rare haplotypes and reduced genotype diversity. During rapid population expansion, common haplotypes are maintained in the population with stable frequency, whereas new haplotypes are constantly introduced by mutation and recombination [41].

Consequently, common haplotypes in the initial population are preserved in the simulated population, but many rare haplotypes will be replaced by new haplotypes. Because mutation has a relatively small impact on common alleles, an increased mutation rate can be used to generate more new haplotypes in the simulated populations without markedly affecting other population properties such as allele frequency and LD patterns. This limitation will become less of a challenge as more human data become available (e.g., from the 1000 Genomes Project [32]).

Because a larger initial population size would reduce the bottleneck effect and help preserve uncommon haplotypes, we combined all independent individuals from 10 populations of the Phase 3 HapMap data set for examples 1, 2, and 4. The sudden population admixture caused long-range admixture LD in the combined initial population. Such admixture LD decayed gradually during evolution and did not lead to elevated long-range LD in the simulated population (Figure 2). Nevertheless, the availability of high-quality sequences of larger samples will allow us to generate population-specific samples and further improve the quality of our simulated data sets.

We evaluated the quality of simulated data sets by comparing allele frequency and LD patterns between the simulated and the HapMap samples. However, we did not attempt to tweak our evolutionary process so that the simulated samples resembled the HapMap sample closely because we aimed to simulate larger populations with more genetic diversity than the founder population and because we wanted to use a realistic evolutionary process so that additional genetic or demographic features could be added. If we consider the method to randomly split and join pieces of chromosomes used by HapSample [14] as a special form of recombination, HapSample could be considered a one-generation forward-time simulation method with magnified recombination rates. Our simulation method would yield results similar to those of the resampling methods if we used an extraordinarily high scaling factor so that all sequences were essentially derived directly from haplotypes of the initial population.

We used a fine-scale genetic map to determine the recombination rate between adjacent markers [35]. This map generally has a higher recombination rate between markers from different haplotype blocks and a lower recombination rate between markers from the same haplotype blocks. Because recombinations happened mostly between existing haplotype blocks, this genetic map helped us retain the haplotype blocks and therefore the LD structure of the founder population. However, due to the relatively short evolutionary time, the type of genetic map does not have a strong influence on the simulated population. For example, there is no discernible difference between LD patterns (e.g. Figure 2) of simulated populations if we use a physical map with a recombination rate of 0.01 per Mbp instead of a genetic map to recombine parental chromosomes during evolution (results not shown).

Our simulation program allows the simulation of arbitrarily chosen selection models with multiple interacting genetic factors and at the mean time allele frequencies at the present generation. If inappropriate parameters are chosen, it is likely that the specified selection model would result in allele frequencies higher or lower than expected so that no valid trajectory of allele frequency could be simulated. If this is the case, our trajectory simulation function will print the average ending frequency for a forward-time simulation or length of trajectory for a backward-time simulation so that the simulation parameters can be adjusted accordingly. This is especially useful if a gene-gene interaction model is used so that marginal selection pressure can interact with allele frequency and drive the allele frequency of DPA in unpredictable directions.

Due to different requirements of different applications, the flexibility of this simulation method is difficult to harness using a traditional single-execution implementation. This is why we divided our simulation approach into three steps and implemented different preprocessing and postprocessing scripts for different applications. These scripts were written in Python and can be executed on any platform where Python and simuPOP are installed. The scripts provide both command line and graphical user interfaces and could be used to simulate samples for applications similar to those described in this paper. For these reasons, it is relatively easy to adapt these scripts for specific applications if different evolutionary processes and/or processing procedures are needed. We provide these scripts through a wiki-based system (page simuGWAS in the "Complete Scripts" section of the simuPOP online cookbook http://simupop.sourceforge.net/cookbook) so that users can share their experiences and improvements of this simulation approach. We expect that more scripts will be contributed as users apply our proposed forward-time simulation approach to a wider variety of GWA studies.

## Conclusions

This paper describes a forward-time simulation algorithm to simulate large populations from an initial population that is created from selected markers of an existing genetic sample. During rapid population expansion, common haplotypes in the initial population are well preserved, whereas new haplotypes are constantly introduced by mutation and recombination, thus add more genetic variations to the simulated population. Compared to other simulation methods, our method simulates samples with existing genetic markers that resemble the human populations well in terms of marker allele frequency and LD structure, with additional flexibility to simulate genomic regions with signals of natural selection. Because of the use of a scripting language design, our implementation of this algorithm can simulate different types of samples with arbitrary disease or quantitative trait models, making it an ideal tool to evaluate the statistical power of a wide variety of statistical gene mapping methods for GWAS.

## Abbreviations

CHB: Han Chinese in Beijing, China; DPA: disease-predisposing allele; DPL: disease-predisposing loci; JPT: Japanese in Tokyo, Japan; LD: linkage disequilibrium; GWAS: genome-wide association study; MKK: Maasai in Kinyawa, Kenya; SNP: single nucleotide polymorphism.

## Authors' contributions

BP conceived of the study, implemented the method and wrote the manuscript. CIA participated in the design of the method, helped to draft and revise the manuscript. Both authors read and approved the final manuscript.

## References

[B1] Sham PC Purcell S Cherny SS Abecasis GR Powerful regression-based quantitative-trait linkage analysis of general pedigrees Am J Hum Genet 2002 71 2 238 253 10.1086/341560 12111667 PMC379157

[B2] Amos CI Krushkal J Thiel TJ Young A Zhu DK de Andrade EBM Comparison of model-free linkage mapping strategies for the study of a complex trait Genet Epidemiol 1996 14 743 748 10.1002/(SICI)1098-2272(1997)14:6<743::AID-GEPI30>3.0.CO;2-O 9433571

[B3] Reich D Patterson N Will admixture mapping work to find disease genes? Phil Trans R Soc B 2005 360 1605 1607 10.1098/rstb.2005.1691 16096110 PMC1609196

[B4] Mehta T Tanik M Allison DB Towards sound epistemological foundations of statistical methods for high-dimensional biology Nat Genet 2004 36 9 943 947 10.1038/ng1422 15340433

[B5] Amos CI Wu X Broderick P Gorlov IP Gu J Eisen T Dong Q Zhang Q Gu X Vijayakrishnan J Genome-wide association scan of tag SNPs identifies a susceptibility locus for lung cancer at 15q25.1 Nat Genet 2008 40 5 616 622 10.1038/ng.109 18385676 PMC2713680

[B6] McCarthy MI Abecasis GR Cardon LR Goldstein DB Little J Ioannidis JP Hirschhorn JN Genome-wide association studies for complex traits: consensus, uncertainty and challenges Nat Rev Genet 2008 9 5 356 369 10.1038/nrg2344 18398418

[B7] Carvajal-Rodriguez A Simulation of Genomes: A review Current Genomics 2008 9 155 159 10.2174/138920208784340759 19440512 PMC2679650

[B8] Wiltshire S Morris AP Zeggini E Examining the statistical properties of fine-scale mapping in large-scale association studies Genet Epidemiol 2008 32 3 204 214 10.1002/gepi.20295 18064636 PMC3076696

[B9] Marchini J Howie B Myers S McVean G Donnelly P A new multipoint method for genome-wide association studies by imputation of genotypes Nat Genet 2007 39 7 906 913 10.1038/ng2088 17572673

[B10] Spencer CC Su Z Donnelly P Marchini J Designing genome-wide association studies: sample size, power, imputation, and the choice of genotyping chip PLoS Genet 2009 5 5 e1000477 10.1371/journal.pgen.1000477 19492015 PMC2688469

[B11] Chai HS Sicotte H Bailey KR Turner ST Asmann YW Kocher JP GLOSSI: a method to assess the association of genetic loci-sets with complex diseases BMC Bioinformatics 2009 10 102 10.1186/1471-2105-10-102 19344520 PMC2678095

[B12] Bochdanovits Z Verhage M Smit AB de Geus EJ Posthuma D Boomsma DI Penninx BW Hoogendijk WJ Heutink P Joint reanalysis of 29 correlated SNPs supports the role of PCLO/Piccolo as a causal risk factor for major depressive disorder Mol Psychiatry 2009 14 7 650 652 10.1038/mp.2009.37 19546850

[B13] Tan HY Callicott JH Weinberger DR Intermediate phenotypes in schizophrenia genetics redux: is it a no brainer? Mol Psychiatry 2008 13 3 233 238 10.1038/sj.mp.4002145 18285755

[B14] Wright FA Huang H Guan X Gamiel K Jeffries C Barry WT Pardo-Manuel F Sullivan PF Wilhelmsen KC Zou F Simulating association studies: a data-based resampling method for candidate regions or whole genome scans Bioinformatics 2007 17785348 10.1093/bioinformatics/btm386

[B15] Li C Li M GWAsimulator: a rapid whole-genome simulation program Bioinformatics 2008 24 1 140 142 10.1093/bioinformatics/btm549 18006546

[B16] Hudson RR Generating samples under a Wright-Fisher neutral model Bioinformatics 2002 18 337 338 10.1093/bioinformatics/18.2.337 11847089

[B17] Mailund T Schierup MH Pedersen CN Mechlenborg PJ Madsen JN Schauser L CoaSim: A flexible environment for simulating genetic data under coalescent models BMC Bioinformatics 2005 6 252 10.1186/1471-2105-6-252 16225674 PMC1274299

[B18] Liang L Zollner S Abecasis GR GENOME: a rapid coalescent-based whole genome simulator Bioinformatics 2007 23 12 1565 1567 10.1093/bioinformatics/btm138 17459963

[B19] Carvajal-Rodriguez A GENOMEPOP: a program to simulate genomes in populations BMC Bioinformatics 2008 9 223 10.1186/1471-2105-9-223 18447924 PMC2386491

[B20] Lambert BW Terwilliger JD Weiss KM ForSim: a tool for exploring the genetic architecture of complex traits with controlled truth Bioinformatics 2008 24 16 1821 1822 10.1093/bioinformatics/btn317 18565989 PMC2732213

[B21] Peng B Amos CI Kimmel M Forward-time simulations of human populations with complex diseases PLoS Genetics 2007 3 e47 10.1371/journal.pgen.0030047 17381243 PMC1829403

[B22] Consortia TH A haplotype map of the human genome Nature 2005 437 7063 1299 1320 10.1038/nature04226 16255080 PMC1880871

[B23] Zollner S von Haeseler A A coalescent approach to study linkage disequilibrium between single-nucleotide polymorphisms Am J Hum Genet 2000 66 2 615 628 10.1086/302766 10677321 PMC1288114

[B24] Wang Y Rannala B In Silico Analysis of Disease-Association Mapping Strategies Using the Coalescent Process and Incorporating Ascertainment and Selection Am J Hum Genet 2005 76 1066 1073 10.1086/430472 15818531 PMC1196444

[B25] McVean GA Cardin NJ Approximating the coalescent with recombination Philos Trans R Soc Lond B Biol Sci 2005 360 1459 1387 1393 10.1098/rstb.2005.1673 16048782 PMC1569517

[B26] Marjoram P Wall JD Fast "coalescent" simulation BMC Genet 2006 7 16 10.1186/1471-2156-7-16 16539698 PMC1458357

[B27] Chen GK Marjoram P Wall JD Fast and flexible simulation of DNA sequence data Genome Res 2009 19 1 136 142 10.1101/gr.083634.108 19029539 PMC2612967

[B28] Chadeau-Hyam M Hoggart CJ O'Reilly PF Whittaker JC De Iorio M Balding DJ Fregene: Simulation of realistic sequence-level data in populations and ascertained samples Bmc Bioinformatics 2008 9 11 10.1186/1471-2105-9-364 18778480 PMC2542380

[B29] Peng B Kimmel M simuPOP: a forward-time population genetics simulation environment Bioinformatics 2005 21 18 3686 3687 10.1093/bioinformatics/bti584 16020469

[B30] Wu CC Shete S Chen WV Peng B Lee AT Ma J Gregersen PK Amos CI Detection of disease-associated deletions in case-control studies using SNP genotypes with application to rheumatoid arthritis Hum Genet 2009 126 2 303 315 10.1007/s00439-009-0672-3 19415332 PMC2992885

[B31] Altshuler D Brooks LD Chakravarti A Collins FS Daly MJ Donnelly P A haplotype map of the human genome Nature 2005 437 1299 1320 10.1038/nature04226 16255080 PMC1880871

[B32] Wise J Consortium hopes to sequence genome of 1000 volunteers BMJ 2008 336 7638 237 10.1136/bmj.39472.676481.DB 18244979 PMC2223041

[B33] Wall JD Przeworski M When did the human population size start increasing? Genetics 2000 155 4 1865 1874 10924481 10.1093/genetics/155.4.1865 PMC1461207

[B34] Ewens WJ Mathematical Population Genetics 2004 Springer

[B35] Myers S Bottolo L Freeman C McVean G Donnelly P A fine-scale map of recombination rates and hotspots across the human genome Science 2005 310 5746 247 248 10.1126/science.1117196 16224025

[B36] Kimura M Weiss GH The stepping stone model of population structure and the decrease of genetic correlation with distance Genetics 1964 49 4 561 576 17248204 10.1093/genetics/49.4.561 PMC1210594

[B37] Hoggart CJ Chadeau-Hyam M Clark TG Lampariello R Whittaker JC Iorio MD Balding DJ Sequence-level population simulations over large genomic regions Genetics 2007 177 3 1725 1731 10.1534/genetics.106.069088 17947444 PMC2147962

[B38] Slatkin M Simulating genealogies of selected alleles in a population of variable size Genetics Research 2001 78 49 57 10.1017/S0016672301005183 11556137

[B39] Slatkin M Linkage disequibrium in gorwing and stable populations Genetics 1994 137 331 336 8056320 10.1093/genetics/137.1.331 PMC1205949

[B40] McVean GAT A Genealogical Interpretation of Linkage Disequilibrium Genetics 2002 162 2 987 991 12399406 10.1093/genetics/162.2.987 PMC1462283

[B41] Peng B Kimmel M Simulations provide support for the common disease common variant hypothesis Genetics 2007 175 1 14 10.1534/genetics.106.058164 17151262 PMC1800600

[B42] Li D Conti DV Detecting Gene-Environment Interactions Using a Combined Case-Only and Case-Control Approach Am J Epidemiol 2009 169 4 497 504 10.1093/aje/kwn339 19074774 PMC2732970

[B43] Voight BF Kudaravalli S Wen X Pritchard JK A map of recent positive selection in the human genome PLoS Biol 2006 4 e87 10.1371/journal.pbio.0040087 16494531 PMC1382018

[B44] Ayodo G Price AL Keinan A Ajwang A Otieno MF Orago ASS Patterson N Reich D Combining Evidence of Natural Selection with Association Analysis Increases Power to Detect Malaria-Resistance Variants 2007 81 2 234 242 17668374 10.1086/519221 PMC1950820

[B45] McVean G The Structure of Linkage Disequilibrium Around a Selective Sweep Genetics 2007 175 3 1395 1406 10.1534/genetics.106.062828 17194788 PMC1840056

[B46] Spencer CC Coop G SelSim: a program to simulate population genetic data with natural selection and recombination Bioinformatics 2004 20 3673 3675 10.1093/bioinformatics/bth417 15271777

[B47] Li M Boehnke M Abecasis GR Joint modeling of linkage and association: identifying SNPs responsible for a linkage signal Am J Hum Genet 2005 76 6 934 949 10.1086/430277 15877278 PMC1196453

[B48] Barrett JC Fry B Maller J Daly MJ Haploview: analysis and visualization of LD and haplotype maps Bioinformatics 2005 21 2 263 265 10.1093/bioinformatics/bth457 15297300

[B49] Knowler W Williams R Pettitt D Steinberg A GM3-5,13,14 and type-2 diabetes-mellitus - an association in american-indians with genetic admixture Am J Hum Genet 1988 43 4 520 526 3177389 PMC1715499

[B50] Pritchard JK Donnelly P Case-control studies of association in structured or admixed populations Theor Popul Biol 2001 60 3 227 237 10.1006/tpbi.2001.1543 11855957

[B51] Devlin B Roeder K Genomic control for association studies Biometrics 1999 55 4 997 1004 10.1111/j.0006-341X.1999.00997.x 11315092

[B52] Price AL Patterson NJ Plenge RM Weinblatt ME Shadick NA Reich D Principal components analysis corrects for stratification in genome-wide association studies Nat Genet 2006 38 8 904 909 10.1038/ng1847 16862161

[B53] Zhu X Luke A Cooper RS Quertermous T Hanis C Mosley T Gu CC Tang H Rao DC Risch N Admixture mapping for hypertension loci with genome-scan markers Nat Genet 2005 37 2 177 181 10.1038/ng1510 15665825

[B54] Smith MW O'Brien SJ Mapping by admixture linkage disequilibrium: advances, limitations and guidelines Nat Rev Genet 2005 6 8 623 632 10.1038/nrg1657 16012528

[B55] Pfaff C Parra E Bonilla C Hiester K McKeigue P Kamboh M Hutchinson R Ferrel R Boerwinkle E Shriver M Population structure in admixed populations: effect of admixture dynamics on the pattern of linkage disquilibrium Am J Hum Genet 2001 68 198 207 10.1086/316935 11112661 PMC1234913

[B56] Weir B Cockerham C Estimating F-Statistics for the Analysis of Population Structure Evolution 1984 38 6 1358 1370 10.2307/2408641 28563791

[B57] Long JC The genetic structure of admixed populations Genetics 1991 127 417 428 2004712 10.1093/genetics/127.2.417 PMC1204369

[B58] Peng B Amos CI Forward-time simulations of non-random mating populations using simuPOP Bioinformatics 2008 24 11 1408 1409 10.1093/bioinformatics/btn179 18417488 PMC2691961

[B59] Tang H Peng J Wang P Risch NJ Estimation of individual admixture: analytical and study design considerations Genetic epidemiology 2005 28 4 289 301 10.1002/gepi.20064 15712363

[B60] Pritchard JK Stephens M Donnelly P Association mapping in structured populations Am J Hum Genet 2000 67 170 181 10.1086/302959 10827107 PMC1287075

[B61] Lander E Schork N Genetic dissection of complex traits Science 1994 265 2037 2048 10.1126/science.8091226 8091226

